# Bacterial Sphingomyelinase is a State-Dependent Inhibitor of the Cystic Fibrosis Transmembrane conductance Regulator (CFTR)

**DOI:** 10.1038/s41598-017-03103-2

**Published:** 2017-06-07

**Authors:** B. B. Stauffer, G. Cui, K. A. Cottrill, D. T. Infield, N. A. McCarty

**Affiliations:** 1 Division of Pulmonology, Allergy/Immunology, Cystic Fibrosis, and Sleep, Department of Pediatrics, Emory + Children’s Center for Cystic Fibrosis and Airways Disease Research, Emory University School of Medicine and Children’s Healthcare of Atlanta, 2015 Uppergate Drive, Atlanta, GA 30322 USA; 2 Molecular and Systems Pharmacology program, Emory University, 201 Dowman Drive, Atlanta, GA 20322 USA

## Abstract

Sphingomyelinase C (SMase) inhibits CFTR chloride channel activity in multiple cell systems, an effect that could exacerbate disease in CF and COPD patients. The mechanism by which sphingomyelin catalysis inhibits CFTR is not known but evidence suggests that it occurs independently of CFTR’s regulatory “R” domain. In this study we utilized the *Xenopus* oocyte expression system to shed light on how CFTR channel activity is reduced by SMase. We found that the pathway leading to inhibition is not membrane delimited and that inhibited CFTR channels remain at the cell membrane, indicative of a novel silencing mechanism. Consistent with an effect on CFTR gating behavior, we found that altering gating kinetics influenced the sensitivity to inhibition by SMase. Specifically, increasing channel activity by introducing the mutation K1250A or pretreating with the CFTR potentiator VX-770 (Ivacaftor) imparted resistance to inhibition. In primary bronchial epithelial cells, we found that basolateral, but not apical, application of SMase leads to a redistribution of sphingomyelin and a reduction in forskolin- and VX-770-stimulated currents. Taken together, these data suggest that SMase inhibits CFTR channel function by locking channels into a closed state and that endogenous CFTR in HBEs is affected by SMase activity.

## Introduction

The Cystic Fibrosis Transmembrane conductance Regulator (CFTR) chloride channel mediates hydration of the airway epithelium and loss-of-function mutations in CFTR lead to cystic fibrosis (CF), a disease characterized by bronchiectasis, mucus plugging, and persistent bacterial infections that induce a chronic inflammatory state^1^. Many CF therapeutics in the pipeline target CFTR with the goal of restoring channel function, but recent findings suggest that the bacterial virulence factor sphingomyelinase (SMase) can diminish CFTR activity in the *Xenopus* oocyte expression system and Calu-3 cells^2–4^. Given the potentially antagonistic effects that bacterial SMase could have on the pharmacological rescue of CFTR channel function, it is important to understand how SMase affects CFTR channel activity.

Lu and coworkers^3, 4^ found that two catalytically distinct orthologs of SMase inhibited CFTR channel function in the *Xenopus* oocyte expression system. The first was SMase C, which liberates phosphocholine to form ceramide, and the second was SMase D, which liberates choline to form ceramide 1-phosphate. Ramu *et al*.^3^ established that inhibition relies on catalysis of sphingomyelin by demonstrating that both an inactive SMase mutant and preparations of wildtype SMase lacking the requisite Mg^2+^ cofactor left CFTR currents unaffected. However, the underlying mechanism by which SMase enzymatic activity inhibits CFTR activity remains unknown. SMase activity leads to multiple biochemical and biophysical changes in cells; specifically, catalysis of sphingomyelin reduces the prevalence of positive charge in the outer-leaflet of the cell membrane^5^, activates various signaling pathways in numerous cell types^6–11^, facilitates the formation of membrane microdomains^12–14^, and induces plasma membrane internalization^15, 16^. It is currently unclear which of these processes may be responsible for inhibition of CFTR chloride currents in the *Xenopus* expression system. Additionally, it is unknown whether SMase can reduce CFTR activity in bronchial epithelial cells.

In the present work, we show that SMase C inhibits CFTR channels expressed in *Xenopus* oocytes without inducing internalization of the channel and present evidence for the requirement of a mobile cytosolic component. Additionally, we show that channel activation state directly affects inhibition rate and that SMase-pretreatment reduces the efficacy of VX-770, a CFTR-targeted therapeutic. Finally, we present evidence that basolateral application of SMase to primary bronchial epithelial cells reduces forskolin (FSK) and VX-770 stimulated currents. These results suggest that SMase inhibits CFTR channels via a novel, state-dependent mechanism and that the presence of SMase in the pulmonary interstitial space may diminish the ability to activate CFTR.

## Results

### Bacterial Sphingomyelinase C Inhibits CFTR in a Non-membrane-delimited Manner

**Figure 1:**
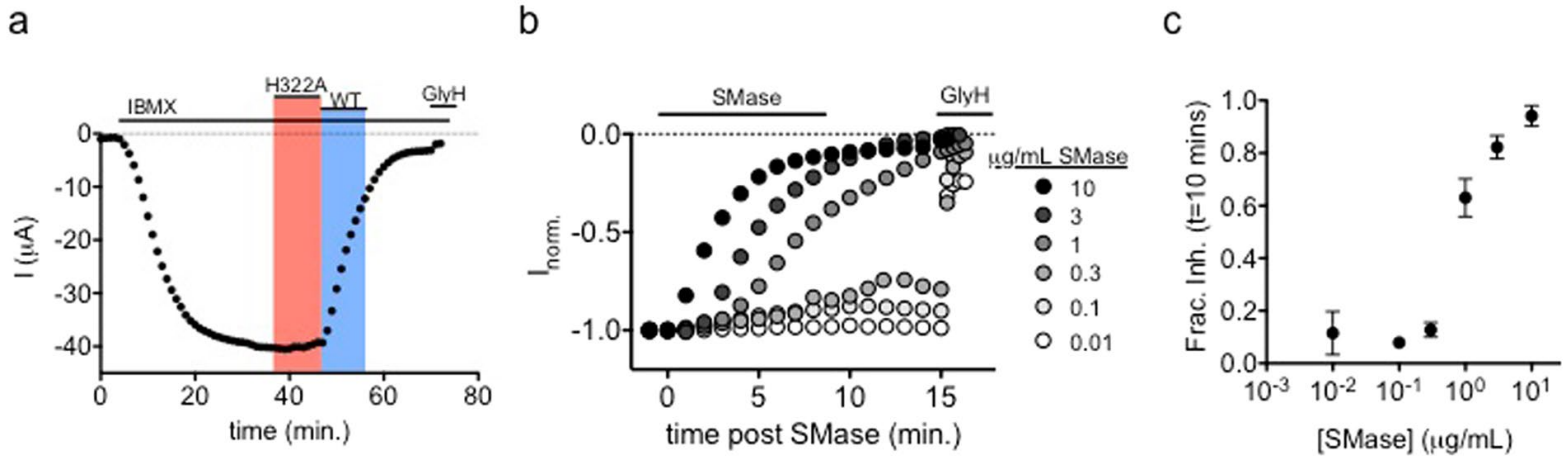
Inhibition results from enzymatic activity of SMase: (**a**) WT-human-CFTR currents elicited with 0.1 mM IBMX and a voltage step to −60 mV were inhibited by 2 μg/mL WT SMase but not by the inactive H322A mutant (n = 6). (**b**) Example traces show that inhibition rate was proportional to SMase concentration. For display, currents were normalized to the amplitude 1 minute prior to application of enzyme. SMase was applied from 0–10 minutes. Sensitivity to GlyH-101 applied at the end of the experiment shows that remaining currents represented uninhibited CFTR channels. (**c**) Summary data showing that fractional inhibition at 10 minutes was proportional to SMase concentration (n = 4–6).

To investigate the effect of *S. aureus* sphingomyelinase C (SMase) on CFTR channel activity, the two-electrode voltage clamp (TEVC) method was used to measure whole-cell CFTR currents in *Xenopus* oocytes after activation of protein kinase A (PKA) mediated signaling by exposure of the cell to 0.1 mM IBMX. After currents reached steady-state activation, cells were exposed to 2 μg/mL wildtype (WT) or enzyme-dead (H322A) SMase in the continuing presence of IBMX. The activity of the purified wildtype enzyme, and loss of activity in the mutant, was confirmed using a fluorometric activity assay (see Supplementary Fig. S1). As shown in Fig. 1a, and consistent with previous observations^3^, CFTR current decreased slowly in the presence of SMase, suggestive of enzymatic activity and possibly the initiation of a signaling cascade that impinged upon CFTR over time, rather than rapid block of the channel pore^17, 18^. Holding the duration of exposure constant at 10 minutes, both the rate and extent of SMase-mediated inhibition of CFTR currents by the active protein were dependent upon SMase concentration (Fig. 1b,c). In these experiments, residual currents were shown to be due to CFTR by their sensitivity to inhibition by GlyH-101^19^.

**Figure 2:**
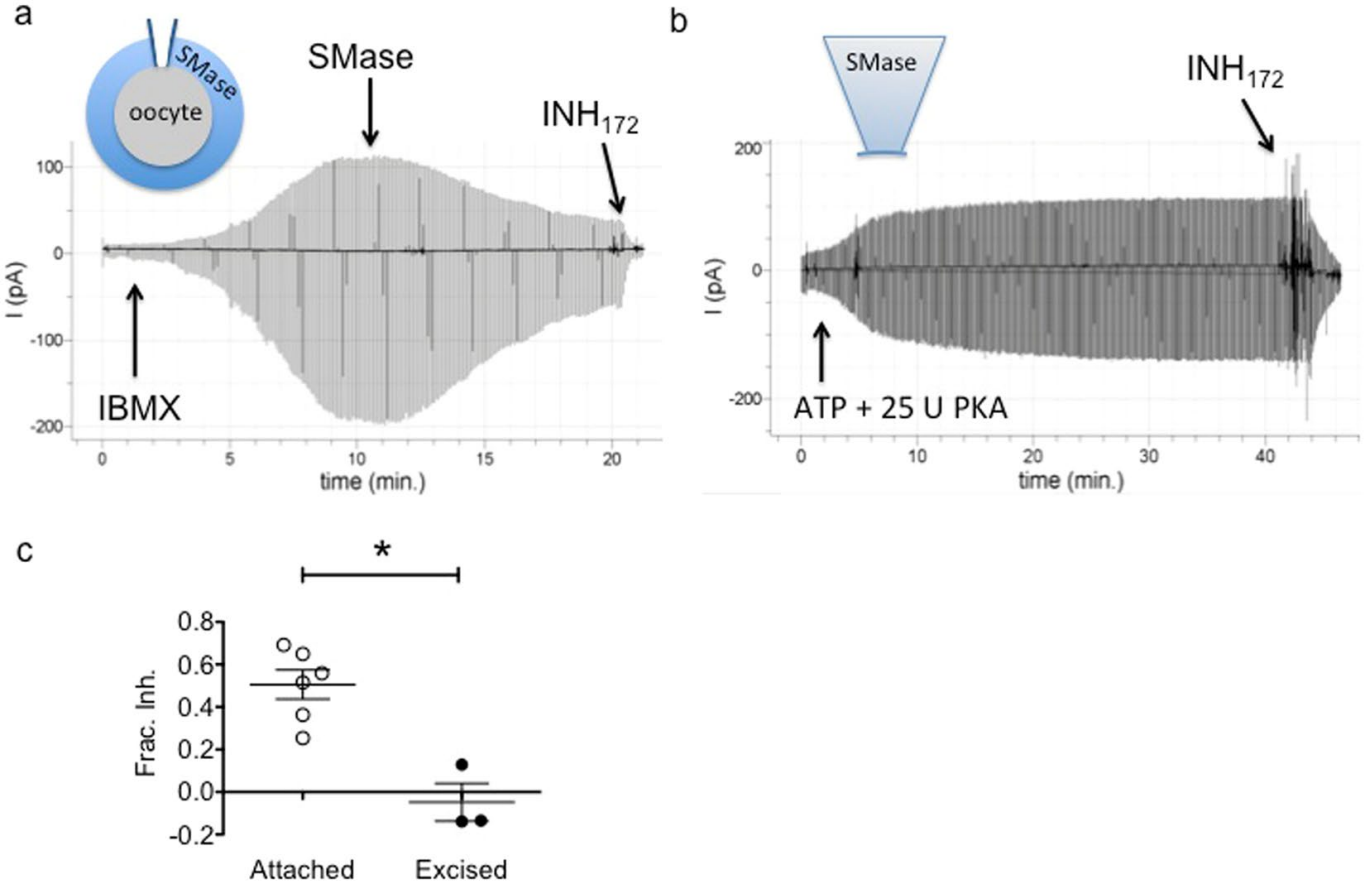
Inhibition is not membrane delimited: (**a**) CFTR currents recorded in the cell-attached configuration after activation with 0.2 mM IBMX were inhibited by 10 μg/ml SMase in the recording chamber suggesting activation of a signaling pathway that impinged upon the channels in the patch. (**b**) CFTR channels activated with 25 U PKA (expected activation of ~50% max)^36^, and recorded in the excised inside-out patch configuration were insensitive to 10 μg/mL SMase backfilled into the recording pipette. (**c**) Summary data showing fractional inhibition of currents in each configuration (p = 0.0021, unpaired t-test).

The results from TEVC experiments as shown in Fig. 1 indicate that inhibition of CFTR by extracellular SMase developed after a brief delay, of duration inversely related to the bath enzyme concentration, and continued after washout of enzyme from the bath solution for concentrations ≥ 1 μg/mL. All of these observations are consistent with the activation of a signaling cascade. To investigate the requirement for cytosolic signaling components, we compared the sensitivity of channels to SMase in two configurations of the macropatch voltage clamp technique^20^ (Fig. 2). In cell-attached macropatch recordings, SMase was added to the solution bathing the entire cell outside of the patch of membrane within the pipette (Fig. 2a). In this configuration, currents reflect the activity of only those channels in the membrane patch protected from direct exposure to SMase by the pipette. Following activation with 0.2 mM IBMX, we found that 10 μg/mL of SMase in the bath led to a reduction in channel currents. We next tested the sensitivity of channels in excised patches. For these experiments, channels were activated by exposure to exogenous PKA at a concentration of 25 U/mL and 1 mM ATP^21^ and directly exposed to 10 μg/mL of SMase backfilled in the pipette-filling solution. In this configuration, we saw no reduction in currents over the course of the 45-minute experiment (Fig. 2b). As before, currents were shown to arise from CFTR by their sensitivity to inhibitor, in this case the cell-permeant CFTR_inh172_^22^. These results, summarized in Fig. 2c, suggest that SMase-mediated inhibition of CFTR channels is not membrane-delimited but instead utilizes mobile signaling components that are lost from the cytoplasmic face of the membrane upon excision of the membrane patch. Additionally, they provide evidence that inhibition does not rely on catalysis of sphingomyelin molecules that may be directly associated with gating CFTR channels.

### SMase-mediated Inhibition of CFTR Does Not Require Retrieval of Channels from the Plasma Membrane

**Figure 3:**
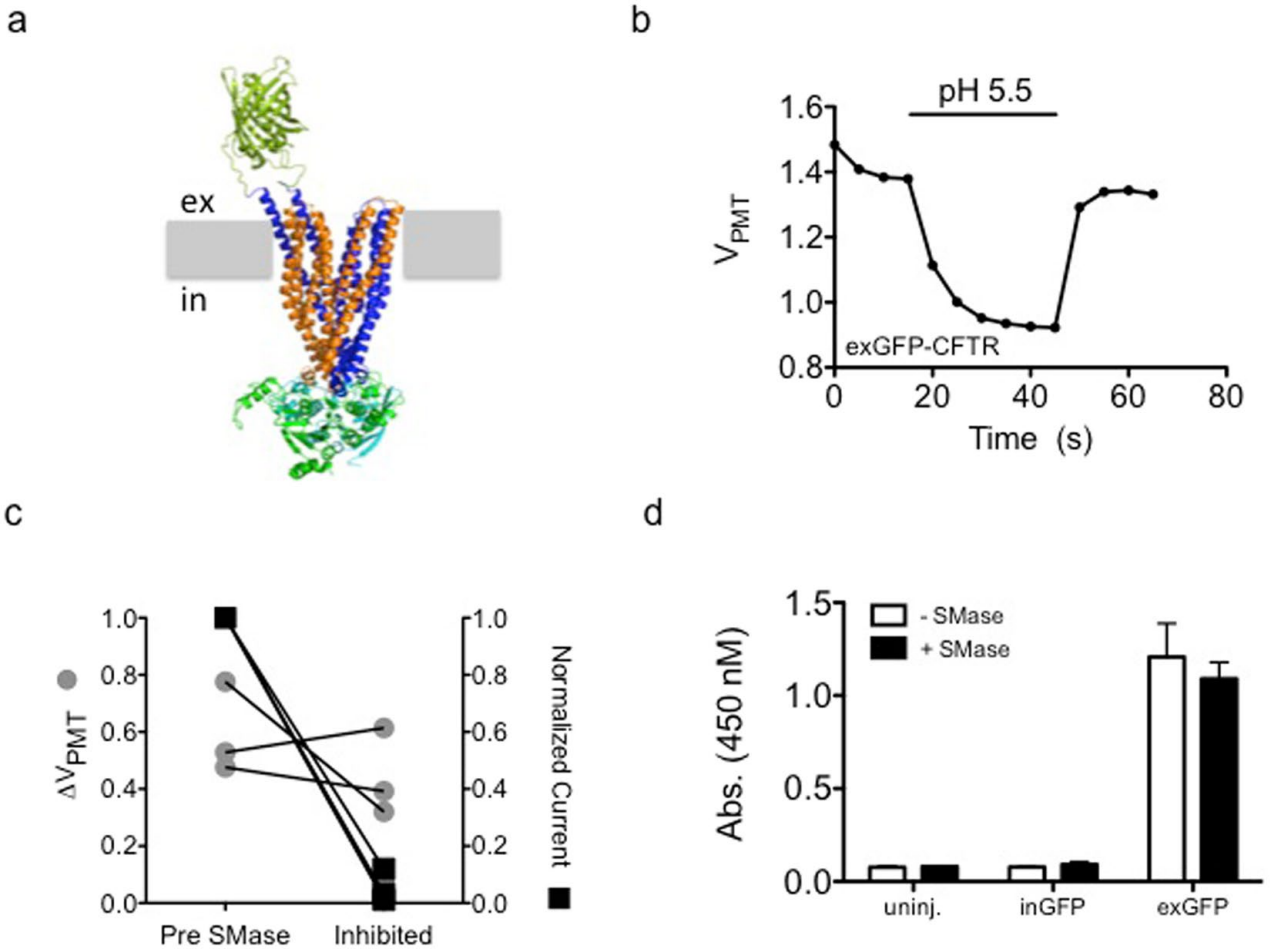
Channel internalization does not underlie SMase-mediated inhibition of CFTR: (**a**) Molecular model of the exGFP-CFTR protein showing the GFP tag on the extracellular side of the plasma membrane (represented in grey). (**b**) Fluorescence measured from oocytes expressing exGFP-CFTR was sensitive to a drop in extracellular pH. Representative background-subtracted voltage from the photomultiplier tube during a brief exposure to pH 5.5 recording solution is shown. (**c**) Treatment with 10 μg/mL SMase for 10 minutes led to inhibition of currents (p = 0.0012) while the pH-dependent fluorescence change was not significantly affected (p = 0.4437, paired t-test). (**d**) In a cell-ELISA experiment, treatment with 10 μg/mL SMase for 10 minutes did not change the accessibility of the extracellular GFP on exGFP-CFTR to an anti-GFP antibody. Uninjected cells and cells expressing inGFP-CFTR showed minimal signal (n = 3).

One potential mechanism by which activation of SMase-induced signaling pathways could lead to loss of CFTR current is by the reduction of channel density in the plasma membrane. SMase has been shown to facilitate internalization in models of membrane repair^16^, so we tested this possibility in three different ways. First, we used a novel assay for CFTR channels resident in the plasma membrane that relies upon the pH sensitivity of a GFP tag in the fourth extracellular loop^23^. Fluorescence of CFTR proteins tagged with this exofacial GFP (exGFP-CFTR; Fig. 3a) was reversibly quenched by perfusion with acidic bath solution (Fig. 3b) while fluorescence of an intracellularly-tagged CFTR (inGFP-CFTR) was unaffected by pH in this timeframe (see Supplementary Fig. S2a,b). Thus, fluorescence quenching is a readout of the abundance of exGFP-CFTR at the plasma membrane. Fluorescence was monitored via a fluorescence microscope coupled to a photomultiplier tube and is shown as a voltage (V_PMT_). To determine whether SMase-mediated inhibition of current corresponded with a proportional reduction in cell surface exGFP-CFTR, we compared the pH-dependent fluorescence change (ΔV) before and after exposure to 10 μg/mL SMase. As shown in Fig. 3c, the pH-dependent change in fluorescence of exGFP-CFTR was not significantly affected by bath SMase despite nearly complete inhibition of the CFTR-mediated current, which was measured simultaneoulsy in the same cells (fractional current inhibition = 0.95 ± 0.056).

We verified this finding using cell-ELISA and biotinylation approaches^24^. For the cell-ELISA experiments the relative surface expression of exGFP-CFTR on oocyte membranes was monitored via binding of a GFP primary antibody and subsequent development with an HRP-conjugated secondary antibody, and the colorimetric TMB substrate. As expected, signal was proportional to the number of exGFP-CFTR expressing cells tested and did not develop when uninjected or inGFP-CFTR cells were used (see Supplementary Fig. S2c). We found that pretreating cells with 10 μg/mL SMase for 10 minutes did not significantly affect the colorimetric signal indicative of exGFP-CFTR at the cell surface (Fig. 3d). Similarly, we found that SMase treatment did not significantly affect labeling of WT-CFTR with the cell impermanent biotinylation reagent sulfo-HNS-SS-biotin (see Supplemental Fig. S3a). Additionally, we found that removal of the PDZ domain from the C-terminal tail of CFTR, which has been shown to mediate internalization of membrane proteins in the oocyte and modulate CFTR plasma membrane stability^25–27^, did not preclude inhibition (see Supplemental Fig. S3b). These results suggest that SMase-mediated inhibition of CFTR does not arise from removal of CFTR proteins from the plasma membrane and instead prevents the normal gating of membrane-embedded channels.

### SMase Inhibits CFTR in a State-dependent Manner

**Figure 4:**
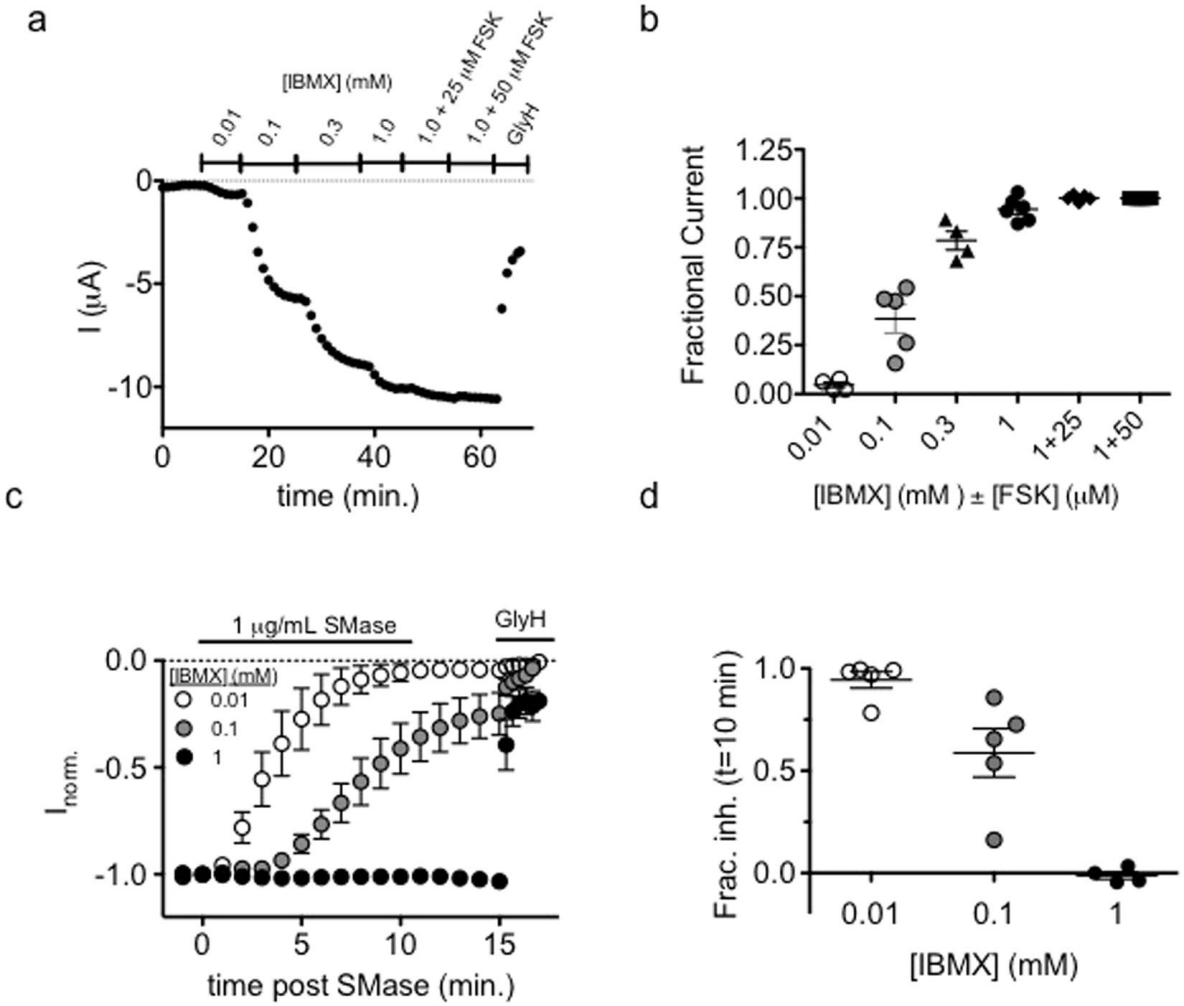
Inhibition is inversely proportional to the level of cAMP-mediated activation of CFTR: (**a**) An example trace shows the graded nature of CFTR channel function where total cellular current increases with increasing IBMX concentration. Maximum current was defined as that which was elicited by 1 mM IBMX + 50 μM FSK. (**b**) Summary data show the fractional current elicited with each activation condition. (**c**) Summary traces show the distinct kinetics associated with inhibition of channels by 1 μg/mL SMase following activation with 0.01, 0.1, or 1.0 mM IBMX (n = 4–5). (**d**) Summary data showing that fractional inhibition at 10 minutes was dependent upon activation condition (p = 0.0003, 1-way ANOVA).

Results to this point suggest that SMase inhibits CFTR activity by affecting channel gating so we next asked whether SMase preferentially affects an open or closed conformation of the channel. CFTR activity is controlled by the phosphorylation of its cytosolic regulatory domain (R-domain) and binding of ATP to the cytosolic nucleotide-binding domains (NBDs)^28, 29^. R-domain phosphorylation by PKA and PKC occurs at several sites, but these sites are not apparently phosphorylated in random order and the consequences of phosphorylation at these sites are not equivalent; in fact, some phosphorylation events are inhibitory while most are stimulatory^30–34^. The cumulative effect of increasing phosphorylation is graded stimulation of CFTR channel activity^35, 36^. In the TEVC configuration, CFTR channel activity can be regulated by varying concentrations of the phosphodiesterase inhibitor IBMX in the presence or absence of forskolin (FSK, Fig. 4a,b). Consistent with previous reports^35, 36^, we found that increasing the IBMX concentration led to a dose-dependent increase in whole-cell (TEVC) currents wherein 0.01, 0.1, and 1.0 mM IBMX resulted in a fractional activity of 0.05 ± 0.01, 0.38 ± 0.07, and 0.94 ± 0.06, relative to 1 mM IBMX + 50 μM FSK (Fig. 4a,b). Graded phosphorylation increases channel open probability (P_o_) to a maximum of ~0.38 under normal conditions without changing open channel unitary conductance^37^.

Lu and coworkers reported that SMase-mediated inhibition of CFTR in oocytes was reduced under strongly activating conditions; however, it was not clear whether this represented a dependence upon CFTR phosphorylation level or CFTR gating state. Specifically they showed that the presence of 50 μM FSK + 1 mM IBMX or heterologously expressed PKA catalytic subunit significantly reduced the magnitude of inhibition compared to oocytes activated with 0.1 mM IBMX^3^. We found similar results and Fig. 4 shows that the rate and extent of inhibition by a ten-minute exposure to 1 µg/mL SMase was inversely proportional to the concentration of IBMX used to activate CFTR (Fig. 4c,d). Cells activated with FSK alone were sensitive to SMase and IBMX did not directly inhibit SMase (see Supplementary Figs S4a and S5) so this is not an off target effect of the activators. One important mechanistic observation reported by Ramu *et al*. was that the phosphorylation-independent CFTR variant lacking the R-domain, split-ΔR**-**CFTR, was sensitive to high extracellular concentrations of SMase, a result we have confirmed^3^ (see Supplementary Fig. 4b). This result suggests that PKA-mediated phosphorylation under strongly activating conditions does not protect CFTR channels from SMase-mediated inhibition via a direct mechanism so we hypothesized that SMase inhibits channels in a state-dependent manner wherein open channels are resistant to inhibition.

**Figure 5:**
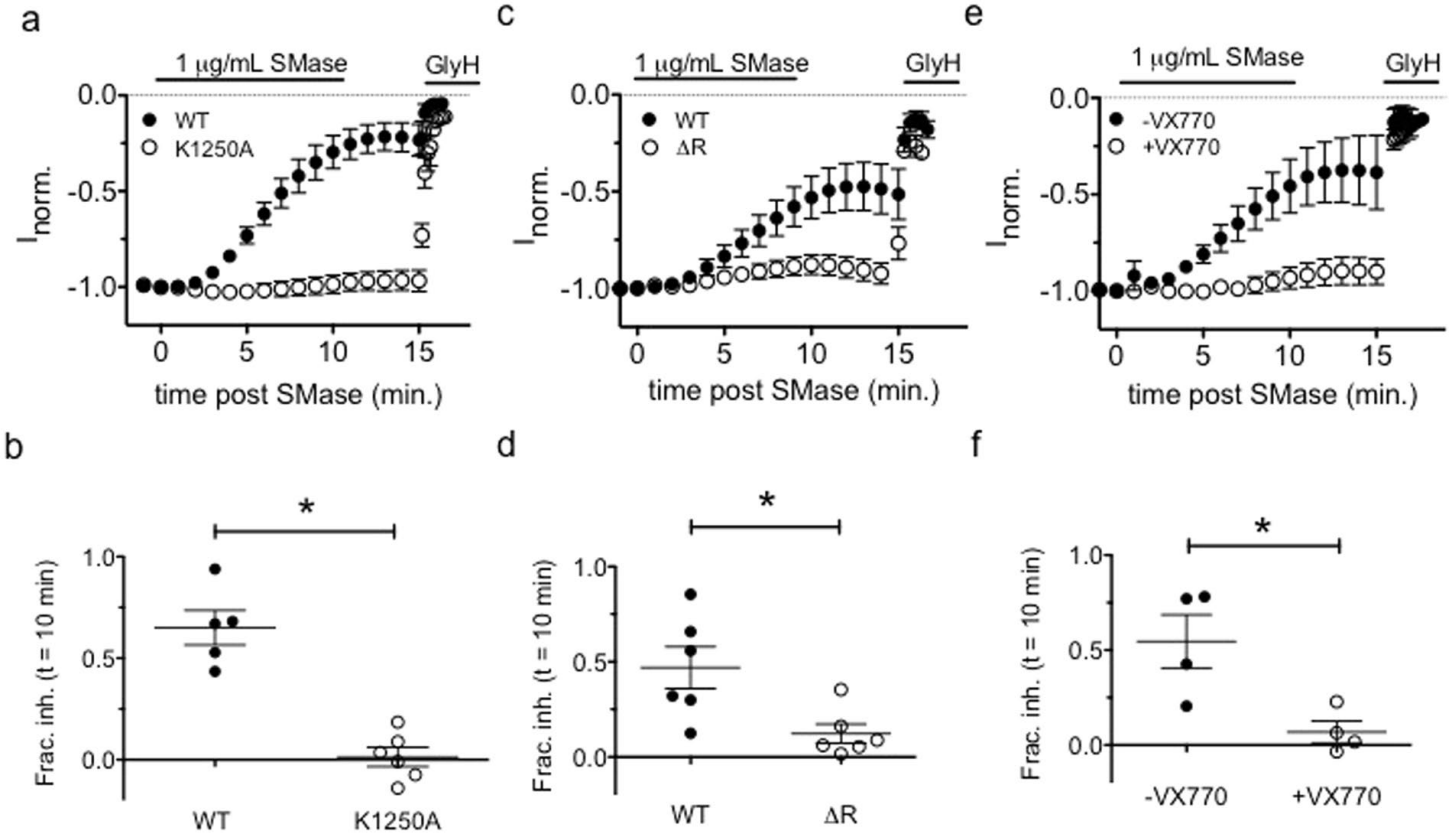
Increased channel activity imparts resistance to SMase-mediated inhibition: (**a**,**b**) The ATP hydrolysis-deficient K1250A-CFTR mutant was resistant to inhibition by 1 μg/mL SMase (p < 0.0001, unpaired t-test). (**c**,**d**) The phosphorylation-independent variant split-ΔR-CFTR was less sensitive to inhibition by SMase than was WT-CFTR in the presence of 0.1 mM IBMX (p = 0.034, unpaired t-test). (**e**,**f**) Potentiation by VX-770 protected CFTR from inhibition by SMase (p = 0.019, unpaired t-test).

To separate the effects of phosphorylation from control of channel gating, we used a series of CFTR channel mutants known to vary in their relative occupancy of the full open state. If our hypothesis is correct, we predict that CFTR mutations that increase channel P_o_ should decrease sensitivity to inhibition by SMase in the presence of equal concentrations of IBMX. To test this hypothesis, we first compared the inhibition rate of WT-CFTR to that of K1250A-CFTR. Lysine1250 plays an important role in ATP hydrolysis at ATP binding-site 2. Mutation to alanine increases the channel P_o_ by decreasing the rate of ATP catalysis and prolonging the mean burst duration^38, 39^. As predicted, we found that K1250A-CFTR was less sensitive to inhibition than WT-CFTR in the presence of 0.1 mM IBMX (mean fractional inhibition = 0.01 ± 0.12 vs. 0.70 ± 0.17 for WT-CFTR, Fig. 5a,b). The difference in sensitivities between these groups did not correspond with different initial current amplitudes (indicative of expression level, see Supplementary Fig. 6), so this result suggests that increasing the fraction of time that channels occupy the full open state decreases the rate of inhibition by SMase.

To ensure that this result did not arise from mutating the ATP binding site, we next compared the sensitivity of WT-CFTR to that of split-ΔR-CFTR. This construct has been shown to have constitutive activity with a relative open probability of approximately half that of highly phosphorylated WT channels, or P_o_ ~0.19^37^. If our hypothesis is correct, we would predict that split-ΔR-CFTR has a different sensitivity to SMase than WT-CFTR when the phosphorylation state of WT-CFTR is such that P_o_ is not 0.19. As expected, we observed constitutive chloride currents in cells expressing split-ΔR-CFTR that was indicative of phosphorylation-independent activity, but also noticed a small increase in current upon exposure of these cells to IBMX (data not shown). While the cause of this effect is not known, potentiation of split-ΔR-CFTR by PKA has been observed in excised inside-out patches from *Xenopus* oocytes^37^ and is indicative of a slight increase in channel P_o_ above 0.19. In our system, activation of WT-CFTR with 0.1 mM IBMX led to a fractional activation of 0.38 ± 0.07 of maximum (Fig. 4a,b), leading to an expected P_o_ of ~0.14^37^. Thus, we predicted that split-ΔR-CFTR (P_o_ > 0.19) is less sensitive to inhibition than WT CFTR activated with 0.1 mM IBMX (P_o_ ≈ 0.14). Indeed, we found WT-CFTR was more sensitive to the inhibitory effect of 1 μg/mL SMase than split-ΔR-CFTR under these conditions with a fractional inhibition of 0.12 ± 0.12 for split-ΔR-CFTR vs. 0.46 ± 0.27 for WT-CFTR (Fig. 5c,d). These data are consistent with the idea that the insensitivity of K1250A-CFTR originates from its high P_o_.

To ensure that the apparent desensitization of K1250A- and split-ΔR-CFTR did not result directly from mutation of the channel, we next used VX-770 (Ivacaftor) as a pharmacological approach to increase channel activity. VX-770 is a highly efficacious potentiator of CFTR that increases P_o_ by increasing the opening rate and stabilizing the open burst duration^40, 41^. Data presented thus far demonstrate that mutations in CFTR that increase gating activity diminish the ability of SMase to inhibit channel activity, so we predicted that potentiation of channel activity by VX-770 would also protect CFTR from SMase-mediated inhibition. To test this, we activated WT-CFTR as before using 0.1 mM IBMX, potentiated currents with 1 μM VX-770 (~EC_90_)^40^ and then treated with SMase. Consistent with results obtained using mutants, WT-CFTR channels that were pretreated with VX-770 were less sensitive to SMase and showed a mean fractional inhibition of only 0.07 ± 0.056 versus 0.54 ± 0.14 in untreated cells studied in parallel (Fig. 5e,f). We verified that this effect is not due to inhibition of SMase by VX-770 (see Supplementary Fig. 7). Taken together, these data provide evidence that SMase is less effective at inhibiting CFTR when channel activity is high and suggest that SMase preferentially affects a closed conformation.

**Figure 6:**
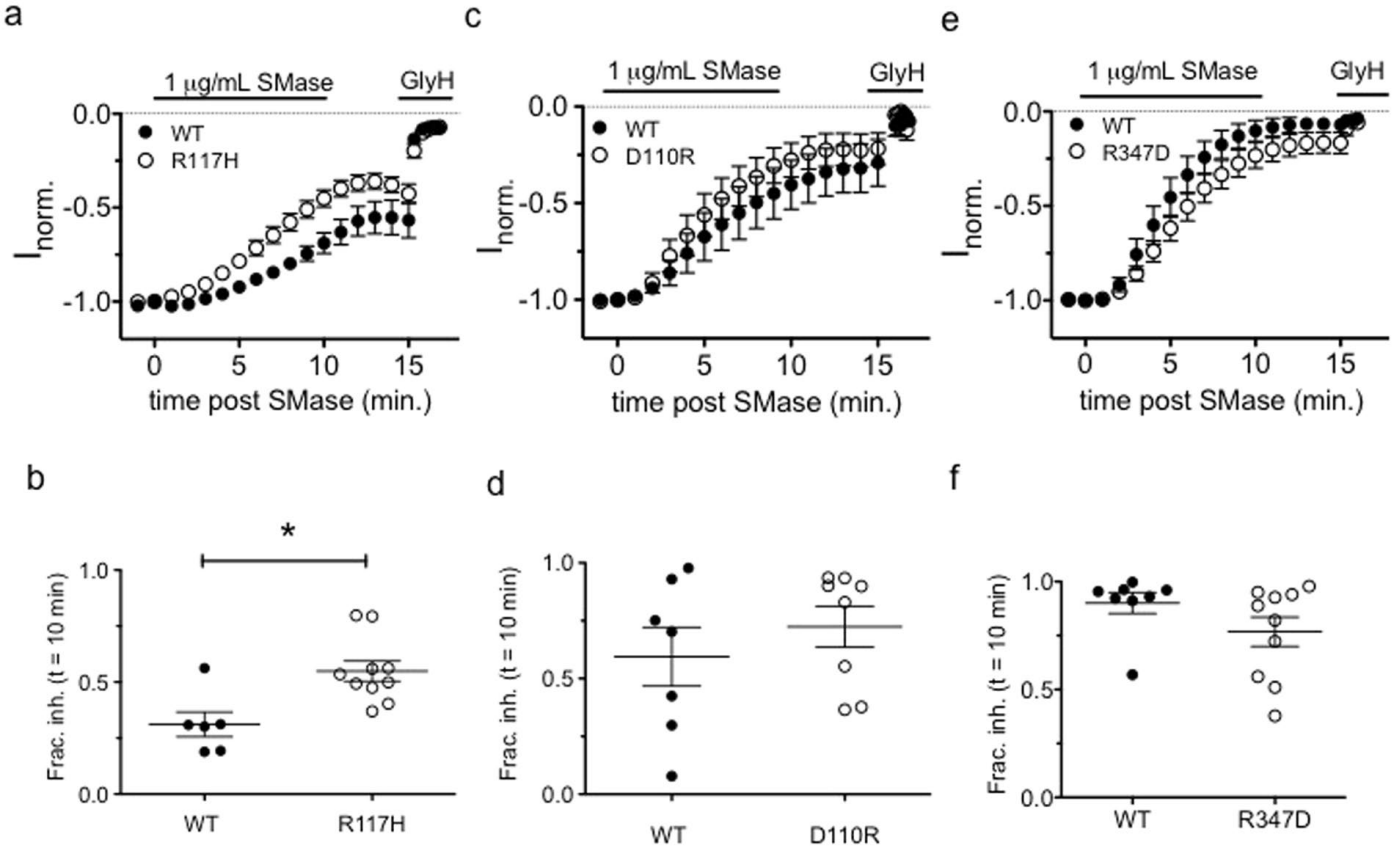
Mutations that disrupt channel pore gating had differential effects on sensitivity to SMase-mediated inhibition: (**a**,**b**) The R117H mutation, which disrupts both pore stability and NBD-dimer stability, increased sensitivity of CFTR to inhibition by SMase (p = 0.0057, unpaired t-test). (**c**–**f**) The D110R and R347D variants of CFTR were inhibited similarly to WT channels (p = 0.348 and p = 0.147, respectively, unpaired t-test).

If SMase targets a closed state of CFTR, increasing the duration of time that channels spend in the affected closed state should increase the sensitivity to inhibition. To test this, we first determined the effect of the CF disease-associated mutation R117H on the sensitivity to SMase. The R117H mutation disrupts gating behavior of CFTR by destabilizing both the pore and the NBD dimer^42^. We found that R117H-CFTR was more sensitive to SMase-mediated inhibition than WT-CFTR studied in parallel with a fractional inhibition of 0.55 ± 0.14 versus 0.31 ± 0.14 for WT (Fig. 6a,b). These data suggest that the R117H mutation increases the occupancy of the closed state that is targeted by SMase. The R117H mutation increases the occupancy of both a “closed permissive” state and an NBD-dedimerized state^42^. Both of these states occur during normal CFTR gating, so to determine whether SMase targets the closed permissive state we compared the sensitivity of D110R-CFTR channels to WT-CFTR channels. Mutations at D110 destabilize the pore and increase the occupancy of the closed permissive state without appearing to impact NBD-mediated gating^43, 44^. We found that D110R-CFTR channels were inhibited at a rate similar to WT-CFTR channels, with a fractional inhibition of 0.72 ± 0.25 versus 0.59 ± 0.34 for WT-CFTR channels studied in parallel (Fig. 6c,d). These data reaffirm the conclusion that the D110R and R117H mutations have distinct deleterious effects on CFTR gating and provide preliminary evidence that SMase targets an NBD-dedimerized closed state. To confirm this conclusion, we tested the sensitivity of R347D-CFTR, which destabilizes the open pore conformation by abolishing a salt bridge required for CFTR pore gating^21, 45^. Like D110R-CFTR, we found that R347D-CFTR was inhibited similarly to WT-CFTR studied in parallel with a fractional inhibition of 0.77 ± 0.21 versus 0.90 ± 0.14 for WT-CFTR (Fig. 6e,f). Taken together, these results suggest that the R117H mutation, but not the D110R or R347D mutations, increases the occupancy of the state that is affected by SMase. Based on published data describing the defect associated with each mutation, these results suggest that SMase targets an NBD dedimerized closed state to inhibit CFTR chloride conductance.

### SMase-mediated Inhibition Abrogates Potentiation by VX-770

**Figure 7:**
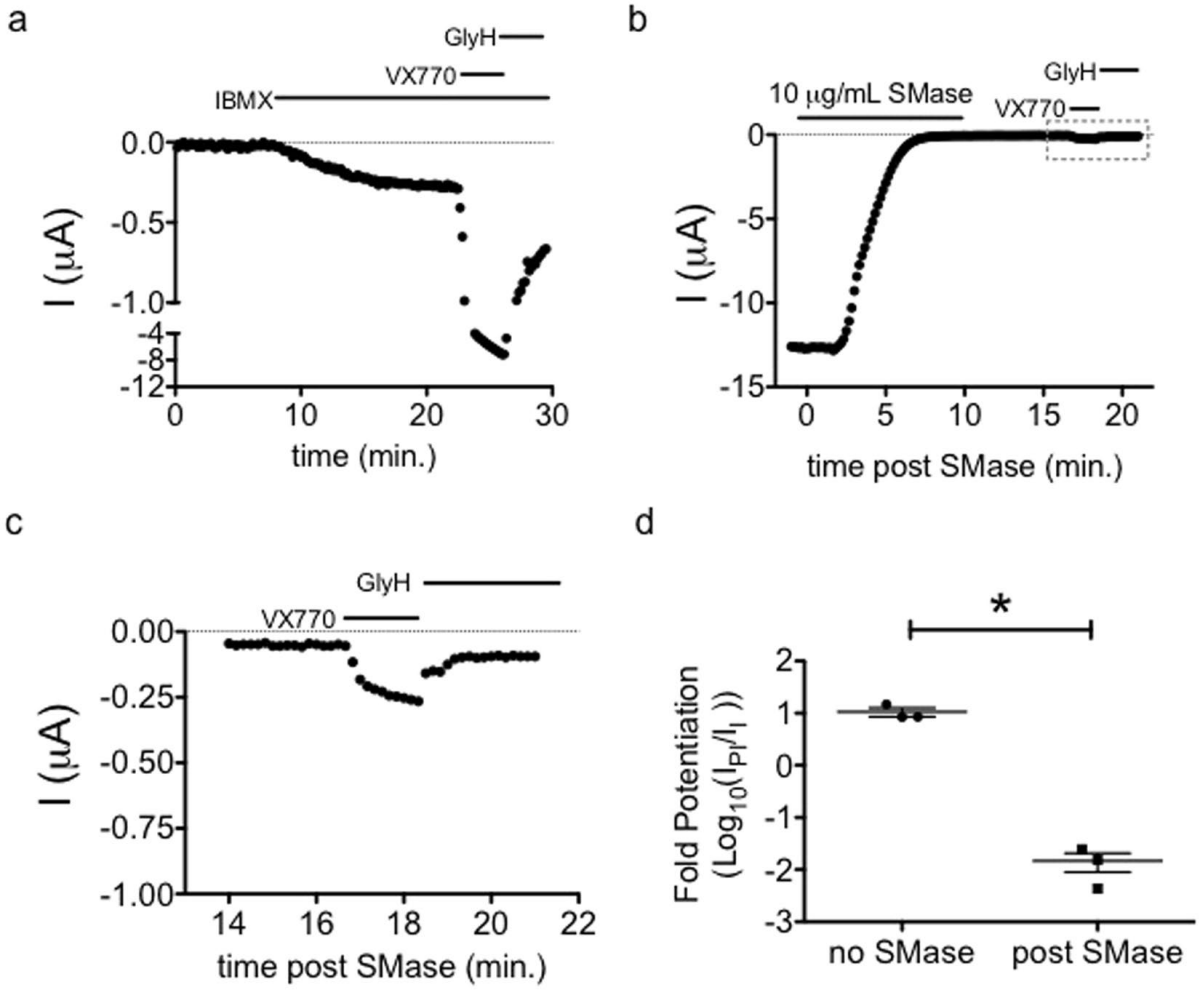
SMase pretreatment diminishes the effect of VX-770: (**a**) Application of VX-770 to WT-CFTR channels activated with 0.1 mM IBMX led to a 10.6 ± 3.6-fold increase in current in 2 minutes. (**b**) An example trace showing that application of 1 μM VX-770 led to a fractional recovery of only 0.01 ± 0.01 when applied after currents were inhibited by SMase. (**c**) Expansion of the area denoted by the grey box in part B shows details of VX-770-mediated potentiation kinetics. (**d**) Summary data showing that the effect of VX-770 on CFTR currents was diminished following inhibition by SMase as compared to IBMX-stimulated currents alone (p = 0.007, unpaired t-test): I_PI_ is the current elicited by VX-770 + IBMX, I_I_ = is the initial current elicited by 0.1 mM IBMX.

Ivacaftor (VX-770) is prescribed to some CF patients over the age of 2 while bacterial infections are often measured in the first year of life, so it may be that pulmonary epithelial cells are exposed to SMase before the patient may take VX-770. To better understand the potential interaction of these two CFTR modulators, we asked whether the inhibition elicited by SMase could be reversed by treatment with VX-770. In the absence of SMase, a two-minute exposure to 1 μM VX-770 potentiated CFTR currents by 10-fold when channels were activated with 0.1 mM IBMX (Fig. 7a,d). However, we found that VX-770 was unable to restore currents following inhibition by SMase. Exposing channels to 1 μM VX-770 restored a small fraction of the current (0.01 ± 0.01, Fig. 7b,c) compared to the original IBMX-stimulated current amplitude. These data demonstrate that the effect of SMase is not immediately reversible by VX-770 and suggest that inhibition by SMase results in channels that are locked closed, effectively removing channels from the druggable pool.

### Basolateral SMase diminishes transepithelial currents in human airway cells

**Figure 8:**
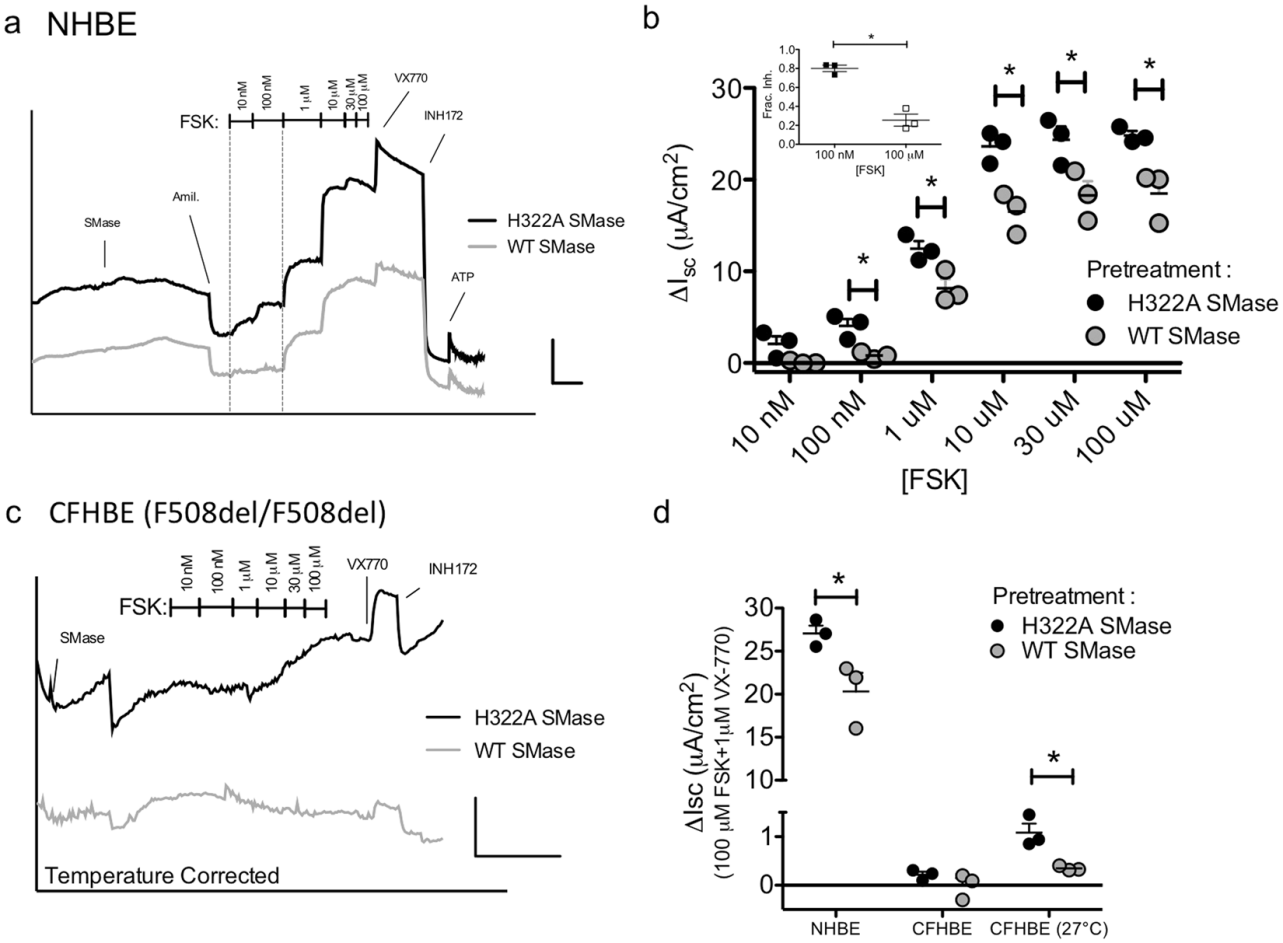
Basolateral treatment with SMase diminishes transepithelial currents in HBEs: (**a**) An example trace showing that pretreatment with basolateral WT SMase diminishes FSK-stimulated transepithelial currents in HBEs expressing WT CFTR. Scale bars: 10 μA/cm^2^, 10 mins. (**b**) Summary data showing significant difference in current amplitudes following basolateral WT SMase (p < 0.0001 for FSK and SMase treatments, 2-way ANOVA; asterisks indicate p < 0.05 for subsequent pairwise t-test analyses). *Inset-*Fractional inhibition is significantly higher in the presence of 100 nM FSK than 100 μM FSK (p = 0.0016, t-test). (**c**) An example trace showing that pretreatment of temperature-corrected CFHBEs with basolateral SMase leads to a similar reduction in maximal current. Scale bars: 2 μA/cm^2^, 20 mins. (**d**) Summary data showing that basolateral treatment with WT SMase reduces currents elicited by 100 μM FSK + 1 μM VX-770 (NHBE p = 0.04, CFHBE-27 °C p = 0.02; t-test).

We next asked whether SMase treatment affects transepithelial currents in primary human bronchial epithelial cells (HBEs). To test this, we modeled our experiment after Ito and colleagues and treated primary HBEs with SMase for 30 minutes before activating currents with FSK^2^. Because SMase inhibited currents more effectively at low activity levels in *Xenopus* oocytes, we tested multiple activation levels of CFTR in HBEs by increasing the FSK concentration in a stepwise fashion. While apical application of SMase did not significantly affect currents (see Supplemental Fig. S8a,b), we found that HBEs treated basolaterally with SMase conducted significantly less current than the control filters pretreated with H322A SMase (Fig. 8a, p < 0.0001). Interestingly, we observed an inverse relationship between FSK concentration and magnitude of inhibition (Fig. 8b). This result is similar to the inverse relationship we observed between CFTR activity and inhibition level in the *Xenopus* oocyte. Importantly, SMase also reduced the current elicited by 100 μM FSK + 1 μM VX-770 by 20 ± 9% (Fig. 8d).

Others have shown that only basolateral SMase treatment affects ion channel function while apical treatment does not^2, 46^; however the mechanism behind this sidedness is not known. Data from early studies of sphingomyelin trafficking in epithelial cells suggest that it is present in both membranes^47^ so we hypothesized that the differential effect was the result of differences in sphingomyelin accessibility. To test this we used an mCherry-tagged sphingomyelin binding protein (mCherry-NT-lysenin) to ask how sphingomyelin abundance and distribution changes following unilateral SMase treatment in primary HBEs^48^. Live cell staining of HBEs with mCherry-NT-lysenin resulted in relatively homogenous staining and solid labeling across the z-axis of the membrane (see Supplementary Fig. 9a). This staining pattern was unchanged following an apical treatment with SMase (see Supplementary Fig. 9b); however, basolateral treatment reduced the basolateral signal and led to a discontinuous and irregular staining pattern (see Supplementary Fig. S9c **)**. This result suggests that only basolateral treatment leads to a marked change in sphingomyelin homeostasis and offers a possible explanation to the unilateral effect of SMase treatment on epithelial ion channel currents.

Finally, we asked whether F508del CFTR homozygous CF human bronchial epithelial cells (CFHBEs) exhibit the same sensitivity to basolateral SMase. To facilitate F508del trafficking to the apical membrane, we incubated the cells at 27 °C for 48 hours^49^. In the absence of low temperature incubation, CFHBE cells did not show any FSK or VX-770 dependent currents (see Supplementary Fig. 8c **)**. As in the NHBEs, we observed significant inhibition of currents following basolateral SMase treatment (Fig. 8c,d). Specifically, currents elicited by 100 μM FSK + 1 μM VX-770 were inhibited by 67 ± 19% (Fig. 8d). A lack of clear stepwise activation with FSK alone made it impossible to quantify inhibition at low FSK concentrations. We saw a similar VX-770-dependence of currents when F508del-CFTR cells were corrected with VX-809 (see Supplemental Fig. S8d). Some of this effect likely also stems from the fact that F508del CFTR is less sensitive to phosphorylation-mediated activation^50^.

## Discussion

Bacterial virulence factors could influence the progression of CF and COPD by altering pulmonary epithelial ion channel physiology. In this study, we began to characterize the inhibitory effect of sphingomyelinase C (SMase) on the chloride channel function of CFTR in two systems. We presented evidence that SMase is a novel gating modifier of CFTR that inhibits membrane embedded channels in *Xenopus* oocytes without relying on catalysis of CFTR-associated sphingomyelin or internalization of the channel. Our results using mutant channels suggest that SMase inhibits currents by affecting closed channels in such a way that they cannot reopen. Finally, we found that basolateral pretreatment with SMase, but not apical pretreatment, decreased FSK- and VX-770-stimulated currents in primary HBEs.

SMase can influence the function of integral membrane proteins either by modulating direct lipid-protein interactions or by altering cellular signaling^5, 46, 51, 52^. Our data suggest that the inhibitory effect of SMase on CFTR function in *Xenopus* oocytes relies on a mobile signaling molecule and does not result from cleavage of annular sphingomyelin. This is supported by the observation that SMase successfully inhibited channels that were isolated from direct exposure to the enzyme in the cell-attached configuration while channels in excised patches were not inhibited when SMase was directly exposed to the channels in the recording pipette (Fig. 2). While our data do not rule out the possibility that inhibition occurs through a direct lipid-CFTR interaction, they do open the extremely interesting possibility that CFTR channel activity may be modulated by an uncharacterized post-translational modification because: (1) the effect does not rely on the R-domain, (2) the formation of ceramide activates multiple signaling cascades and (3) previous reports show that CFTR can be modified in multiple functionally-uncharacterized loci^6–8, 10, 11, 53–55^.

It remains to be seen which sphingolipid is responsible for the observed inhibition by SMase. Given that ceramide is expected to be the most abundant product in the membrane, it is the most likely effector. However, Ramu and coworkers began to test this possibility in oocytes by adding C8-ceramide to the recording chamber. After observing no change in CFTR current, they concluded that ceramide signaling was not responsible for inhibition^3^. One potential issue with this approach stems from the observation that ceramide-mediated signaling can be highly dependent on acyl chain length^56^ and C8-ceramide makes up a small fraction of ceramide in the *Xenopus* oocyte with the predominant species being long chain C16 and C18 ceramides^57^. Ceramide can also be subsequently metabolized into other active signaling molecules to influence cellular physiology, for example ceramide 1-phosphate, sphingosine, and sphingosine 1-phosphate, so it is possible that another sphingolipid is responsible for the observed inhibition. This complex series of metabolic transformations ultimately leads to “metabolic ripple effects,” as described by Hunnan and coworkers^58^, and elucidating the sphingolipid species responsible for SMase-mediated inhibition of CFTR will provide valuable mechanistic insight into this novel regulatory mechanism.

Ramu and coworkers provided some intriguing mechanistic information that suggested SMase was a novel gating modifier of CFTR and, indeed, our data provide direct evidence that SMase is a state-dependent inhibitor of CFTR. This is supported by the observation that sensitivity to inhibition is influenced by channel gating kinetics. The K1250A mutation, which slows channel closure, and pretreatment with VX-770 imparted resistance against the inhibitory effect of SMase (Fig. 5). Additionally, the R117H mutation, which accelerates channel closure in two distinct ways^42^, increased the sensitivity of channels to inhibition by SMase. Our observation that D110R and R347D-CFTR^21, 44^ were inhibited similarly to WT leads us to hypothesize that SMase affects an NBD-dedimerized closed state of CFTR. Whether sensitivity to SMase requires loss of ATP or ADP from the ATP-binding sites following NBD dedimerization remains to be determined.

Our observation that basolateral SMase diminishes FSK-stimulated currents in primary HBEs suggests that sphingolipid metabolism is functionally linked to CFTR in mammalian epithelial cells as well as in *Xenopus* oocytes. Numerous other studies have linked CFTR dysfunction to sphingolipid metabolism in mammalian cells through multiple mechanisms and these results add additional evidence for conserved signaling networks^59–62^. We observed more robust inhibition at low activation levels, which supports the idea that inhibition occurs at CFTR because apical chloride conductance is rate-limiting at low/medium levels of anion secretion while potassium channels become limiting at high levels of secretion^63^. Additionally, CFTR is the primary regulator of this conductance in our HBE line as evidenced by its sensitivity to INH_172_. Future pharmacological studies will dissect this effect further.

Similar to results in cell lines, we found that only basolateral application of SMase affected FSK-stimulated currents in polarized primary epithelial cells^2, 46^. We also presented evidence that this unilateral sensitivity results from differential sensitivities in sphingomyelin homeostasis (see Supplemental Fig. 9). Whether bacterial SMase can access the basolateral side of epithelial cells remains to be seen but recent reports suggest that a CF-derived goblet cell line has dysfunctional tight junction trafficking that might enable the movement of bacterial proteins across the epithelium^64^. Grassme and colleagues have also shown that lymphocytes translocate acid-SMase to the plasma membrane following activation, which opens up the possibility that transmigrating immune cells might influence the local epithelial ion channel function through this mechanism^65^.

Numerous observations suggest that SMase is a functionally conserved pathogenic virulence factor. For example, SMase gets upregulated in *S. aureus* upon instillation into the murine lung^66^, likely through the mobilization of an inactivating phage^67, 68^, where it facilitates inflammatory lung damage^69^. Multiple groups have shown that *B. cereus* SMase decreases macrophage phagocytosis and increases mortality in murine and ovine models of infection^70–72^. Wargo and coworkers implicated *P. aeruginosa* SMase (PlcH) in the reduction of lung function associated with bacterial instillation, though this effect cannot be directly linked to SMase activity because PlcH also hydrolyzes phosphatidylcholine^73^.

What evolutionary benefit might stem from SMase-mediated inhibition of CFTR by bacteria ? One possibility is that inhibition of CFTR may blunt early immune responses to inhaled pathogens. Support for this idea lies in the observation that CFTR KO models are more susceptible to bacterial infections^61, 74^. How CFTR mediates innate immunity is still an active area of research and multiple hypotheses have been proposed. First, as the primary regulator of ASL depth, mucocilliary clearance of inhaled pathogens depends on CFTR function and it is possible that a reduction of CFTR activity might slow the movement of pathogens up the mucocilliary ladder^63, 75^. Secondly, CFTR has been implicated in internalization of some bacteria by epithelial cells, as well as their subsequent apoptosis^76, 77^. Therefore, inhibiting CFTR could slow bacterial endocytosis and prolong their residence time in the airways. Finally, CFTR-mediated bicarbonate secretion has been linked to proper pH homeostasis in the airway of CF model organisms^78, 79^. The acidification of ASL that is associated with loss of CFTR function has been shown to diminish the ability of small antimicrobial peptides to adequately kill bacteria^78^. These results suggest that understanding the impact of SMase activity on CFTR function may be relevant to the treatment of CF.

## Methods

### Source of reagents

L-15 media was from Gibco. GlyH-101 was from EMD Millipore. All other reagents were obtained from Sigma-Aldrich.

### Recombinant Protein purification

The gene encoding *S. aureus* SMase was amplified out of ATCC strain 29213 and inserted into the pET28b inducible expression vector (Novagen) using the Gibson Assembly Method (New England BioLabs) according to the manufacturer’s instructions. *S. aureus* genomic DNA was isolated using the Wizard Genomic DNA purification kit (Promega). The SMase N-terminal signal sequence (AAs 1-34) was removed to prevent secretion and simplify purification. Primers to amplify SMase were as follows: Forward-CTTTAAGAAGGAGATATACCATGGAATCTAAGAAAGATGATACTG; Reverse-GCTCGAGTGCGGCCGCAAGCTTTTTACTATAGGCTTTGATTGG. Primers to amplify pET28b were as follows: Forward-AAGCTTGCGGCCGCACTCGAGC; Reverse-CCATGGTATATCTCCTTCTTAAAG. The inactivating H322A mutation was introduced using QuikChange mutagenesis (Agilent). The expression host was BL21(DE3) *E. coli* and protein production was induced with 0.1 mM IPTG at 16 °C overnight. Cells were pelleted, resuspended in PBS + bacterial protease inhibitor cocktail (Goldbio), and lysed using a French press. Insoluble material was cleared via centrifugation at 10,000 × g for 10 minutes. Protein was purified from the cleared lysate using “cOmplete” His-Tag Purification Resin (Roche) and Poly-prep chromatography columns (Bio-Rad) according to the Roche protocol. Imidazole was removed via dialysis. Eluate was stored in 250 mM sodium phosphate (final concentration)/50% glycerol at 4 °C. SMase C activity was quantified using the Amplex Red sphingomyelinase assay described below. Experiments testing the effect of SMase on CFTR variants were always matched with same-day controls using WT-CFTR. We followed a detailed description of mCherry-NT-lysenin purification that had been published previously^80^.

### Fluorometric SMase assay

The fluorometric assay kit was purchased from Invitrogen (MP12220) and set up according to the manufacturer’s instructions. Briefly, the indicated amount of purified SMase protein was mixed with reaction buffer to a final volume of 100 μL and the reaction was initiated by injecting an equal volume of the reaction mixture containing sphingomyelin, Amplex Red, alkaline phosphatase, choline oxidase, and horseradish peroxidase for a final volume of 200 μL. The assay indirectly measures SMase activity via enzyme-dependent oxidation of the phosphocholine (ChoP) product to H_2_O_2_, and subsequent formation of resorufin from Amplex Red. In all experiments, equimolar ChoP was used as a positive control to determine the maximum signal and the useful range (see Supplementary Fig. S5). It also ensured that our treatments (e.g., exposure to VX-770) did not alter the assay independent of SMase (see Supplementary Fig. S7).

### Preparation of oocytes and cRNA

The mutants used in this study were prepared using the QuikChange site-directed mutagenesis method (Agilent Technologies). Mutant constructs were verified by sequencing across the entire open reading frame before use. All cRNAs for electrophysiology experiments were prepared from a construct encoding WT-CFTR in the pGEMHE vector provided by D. Gadsby (The Rockefeller University, New York, NY) using the mMessage mMachine T7 *in vitro* transcription kit (Ambion). *Xenopus* oocytes were injected with a range of 0.5–15 ng CFTR cRNAs and were incubated at 17 °C in modified Leibovitz’s L-15 media plus 21.8 mM HEPES, pH 7.5, penicillin, and streptomycin^44^. Recordings were made 18–72 h after the injection of cRNAs. Methods of animal handling are in accordance with the National Institutes of Health guidelines, and the protocol was approved by the Institutional Animal Care and Use Committee of Emory University.

### Electrophysiology

In all cases data were acquired using pClamp software and analyzed using Clampfit. For Two Electrode Voltage Clamp (TEVC), CFTR currents were recorded using a Geneclamp 500B amplifier equipped with a virtual ground and pClamp 10.2 software (Axon). Electrodes were pulled from borosilicate glass with a filament and filled with 3 M KCl. Resistances were 0.2–0.5 MΩ. Bath solution was standard ND96 containing (in mM): 96 NaCl, 2 KCl, 1 MgCl_2_, and 5 HEPES, pH 7.5. Cells were held at V_m_ = −20 mV and currents were elicited at 0.1 Hz using a 500 ms ramp from V_m_ = −60–0 mV. Data were acquired at 1 kHz and software-filtered at 100 Hz. Current amplitudes reported in the Results section were measured at V_m_ = −60 mV. SMase was diluted in ND96 at room temperature immediately before application and applied at 2 mL/min using a gravity-fed perfusion system into a recording chamber holding approximately 300 μL of solution. GlyH-101 was applied at 30 μM to ensure that residual current was CFTR. To control for batch-to-batch variation in sensitivity of cells to SMase, equal numbers of cells expressing WT and mutant CFTR were utilized each week. For excised macropatch experiments, the vitelline membrane was removed and the cell was submerged in a recording solution containing (in mM): 150 NMDG-Cl, 1.1 MgCl_2_, 2 Tris-EGTA, and 10 TES, pH 7.4. After excision, inside-out patches were exposed to recording solution containing 1 mM ATP and 25 U/mL PKA. Pipette solution contained 150 NMDG-Cl, 5 MgCl_2_, and 10 TES, pH 7.4. For cell-attached macropatch recordings, a window was generated in the vitelline membrane and the cells were submerged in the pipette solution outlined above; the recording pipette accessed the plasma membrane via the tear in the vitelline membrane. Recording solution in the chamber was then replaced with IBMX-containing solution to activate CFTR channels. In both cases, currents were elicited with a 0.6 Hz ramp from V_m_ = −100 mV to + 100 mV.

### Voltage clamp fluorometry

For real-time measurement of the plasma membrane localization of CFTR via voltage clamp fluorometry, oocytes were injected with cRNA encoding CFTR that was tagged with GFP, either on the intracellular N-terminus^81^ or within extracellular loop 4^82^. Acidic ND96 solution (pH 5.5) was buffered with 10 mM MES instead of HEPES, but was otherwise prepared as described for ND96 above. The voltage clamp fluorometry rig was built similarly as described by Pless^83^. Briefly, fluorescence from oocytes was measured via a photomultiplier tube (Hamamatsu no. H9306-02), and the signal was digitized into pClamp 9 in parallel with the ionic current. The photomultiplier tube was controlled by an Oriel power supply (Newport no. 70703) and set to 650 volts via a 10 kOhm potentiometer. To minimize photobleaching, excitation from the halogen lamp (Lumen Dynamics Xcite no. 120Q) was reduced via a neutral density filter (Thorlabs no. NE03B) and shuttered via TTL pulses from pCLAMP 9 to a shutter driver (Vincent Associates no. VCM-D1) and Uniblitz shutter (Vincent Associates no. 9002-0212), such that the oocytes were exposed to excitation for 100 ms/s. Fluorescence measurements were performed before activation of CFTR currents (to determine the initial level of CFTR at the plasma membrane) and 5 minutes after the end of SMase exposure (to determine the level of CFTR at the plasma membrane post inhibition).

### *Xenopus* oocyte cell-ELISA

Oocytes were washed with ND96 solution and 20 cells were used for each replicate in Fig. 3d. Cells were treated with 10 μg/mL SMase in ND96 for 10 minutes, treatment solution was removed, and cells were fixed in 4% paraformaldehyde in PBS for 20 minutes at room temperature. Oocytes were blocked from nonspecific binding of antibody using 10% bovine calf serum in PBS for 45 minutes. Anti-GFP antibody (Novus #NB600-308) was diluted 1:500 in blocking solution and cells were treated for 2 hours at room temperature. Primary antibody was removed, cells were washed 2 times for 10 minutes each, and a 1:1000 dilution of HRP-conjugated secondary in blocking solution was added to the cells (Cell Signaling #7074). TMB-ELISA substrate (Biolegend) was used to develop signal. Signal was read after a 20-minute incubation at room temperature.

### *Xenopus* oocyte surface biotinylation

Cells were injected with 1 ug of WT-CFTR RNA and incubated at 17 °C until the day of the experiment (4 days). Each treatment group was made up of 15 oocytes and treatments were performed in triplicate. Streptavidin resin was washed in PBS and incubated in oocyte solubilization buffer containing 5% BSA until needed (Solubilization buffer: 20 mM TRIS, 10% v/v glycerol, 5 mM EDTA, 1% w/v Na^+^ deoxycholate, 1 mM PMSF; pH 6.8). Cells were moved to 35 mm dishes, washed 2 times in ND96, and then treated with the appropriate enzyme (WT-SMase or H322A-SMase). After 10 minutes at room temperature, solution was removed and cells were washed 3 times in PBS without Ca^2+^ and Mg^2+^ to quench the SMase activity. Sulfo-NHS-SS-Biotin was added to a final concentration of 0.5 mg/mL and the cells were incubated at room temperature for 30 minutes. After labeling, the biotinylation solution was removed and the reaction was quenched with 3 TBS washes. Oocytes were moved to ice-cold homogenization buffer (20 mM TRIS, 5 mM MgCl_2_, 5 mM NaHPO_4_, 1 mM EDTA, 80 mM sucrose, 1 mM PMSF; pH 7.4), and lysed with fine scissors and serial trituration. Homogenate was then centrifuged at 16,200 × g for 30 minutes at 4 °C to pellet membranes and separate yolk. Supernatant was then aspirated, the pellet was resuspended in solubilization buffer, and the samples were centrifuged 16,200 × g for 30 minutes at 4 °C. A small fraction of the supernatant was removed as the “total CFTR” control. The remaining supernatant was moved to a new tube, mixed with preblocked streptavidin beads, and rotated for 2 hours at 4 °C. After binding, beads were separated using a centrifugation filter, washed with 20 volumes of PBS, and eluted in Laemmli buffer. Eluate was incubated at 37 °C for 30 minutes and then separated on a precast 4–20% gradient polyacrylamide gel. Protein was transferred onto PVDF membranes, blocked with 5% nonfat dry milk, probed with a CFTR primary antibody (UNC #596), and an anti-mouse HRP secondary antibody. Membranes were developed using luminescent HRP substrate and imaged on a BioRad Gel Doc imager.

### Ussing Chamber Recording

Plated primary bronchial epithelial cells (HBEs) were obtained from the CF@LANTA RDP Experimental Models Core and maintained at an air/liquid interface with basolateral “Emory ALI” media (developed by and purchased from the Core). All equipment was sourced from Physiologic Instruments: Amplifier-VCC-MC6; electrodes-P2020-S; Chambers-EM-RSYS-2; Sliders-P2302T. On the day of experimentation, electrode tips were prepared according to the manufacturer’s protocol. Solutions contained (mM): basolateral-115 NaCl, 5 KCl, 1 MgCl_2_, 2 CaCl_2_, 10 glucose, 10 HEPES, 25 NaHCO_3_; apical-115 NaGluconate, 5 KCl, 1 MgCl_2_, 4 CaCl_2_, 10 glucose, 10 HEPES, 25 NaHCO_3_. The voltage offset and fluid resistance compensation were set according to manufacturer’s instructions with a blank filter in the chamber. Both solutions were bubbled with a 95/5 mixture of O_2_/CO_2_ and heated to 37 °C. Data were acquired using Acquire and Analyze software.

### Immunocytochemistry and Confocal Microscopy

Primary HBEs were washed once in PBS + Ca^2+^/Mg^2+^ and treated either apically or basolaterally with 10 μg/mL SMase for 10 minutes at 37 °C. Primary HBEs were stained with mCherry-NT-lysenin as described previously^84^. Specifically, purified mCherry-NT-lysenin was added bilaterally after the SMase treatment and the cells were incubated for 10 minutes at 37 °C. Cells were fixed with 4% PFA + 0.2% glutaraldehyde at room temperature for 30 minutes and the filters were then mounted with a DAPI-containing solution onto glass coverslips. Immunofluorescence Z-stack images of mCherry-NT-lysenin and DAPI were collected at 0.49 micron steps on an inverted Olympus FV1000 Confocal Microscope and analyzed by ImageJ v1.50i (NIH).

### Statistics and data analysis

Data were compared using an unpaired t-test, paired t-test, 1-way ANOVA, or 2-way ANOVA analysis as indicated in the figure legends. In all cases, error bars represent S.E.M. and differences were considered significant when p ≤ 0.05.

## Electronic supplementary material

Supplementary information
