# Supplementary material for: Bacterial Sphingomyelinase is a State-Dependent Inhibitor of the Cystic Fibrosis Transmembrane conductance Regulator (CFTR)
Source: Sci Rep. 2017 Jun 7;7:2931. doi: 10.1038/s41598-017-03103-2 (PMC5462758; doi:10.1038/s41598-017-03103-2)
Supplement: Supplementary file 1 — Supplementary information [file 41598_2017_3103_MOESM1_ESM.pdf]

Supplementary information for, “Bacterial Sphingomyelinase is a State-dependent Inhibitor of the Cystic Fibrosis Transmembrane conductance Regulator (CFTR).”

BB Stauffer, G Cui, KA Cottrill, DT Infield, and NA McCarty

Supplementary Fig. S1

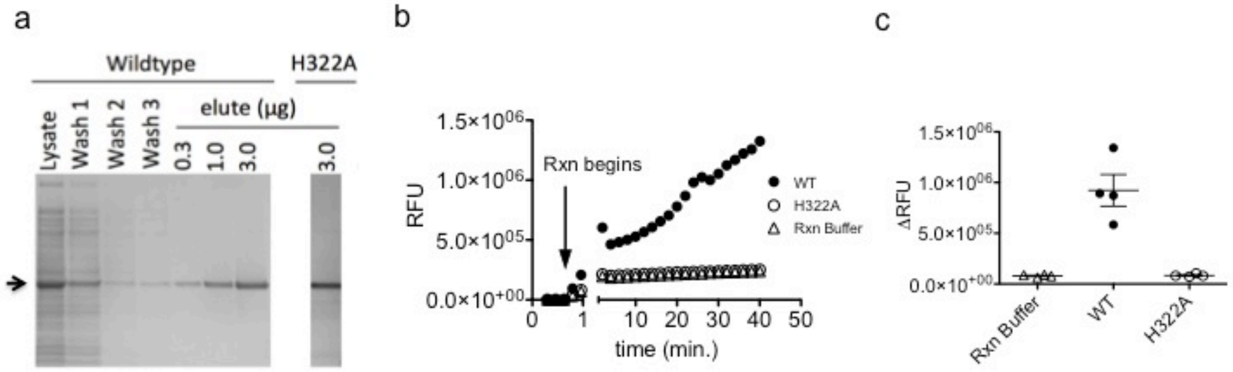

Supplementary Figure S1 – Purification and characterization of recombinant WT and H322A SMase: (a) A Coomassie stained polyacrylamide gel showing purification of both the wild-type and enzyme-dead mutant (H322A) SMase proteins at the expected size (37 kDa). (b) Activity of 100 ng of the WT protein was verified using the commercially available fluorometric SMase assay (see methods) and is apparent by the time-dependent increase in fluorescence. An equal amount of H322A protein showed no activity. (c) Cumulative data showing activity of the WT protein relative to reaction buffer and the H322A protein at 40 minutes.

Supplementary Fig. S2

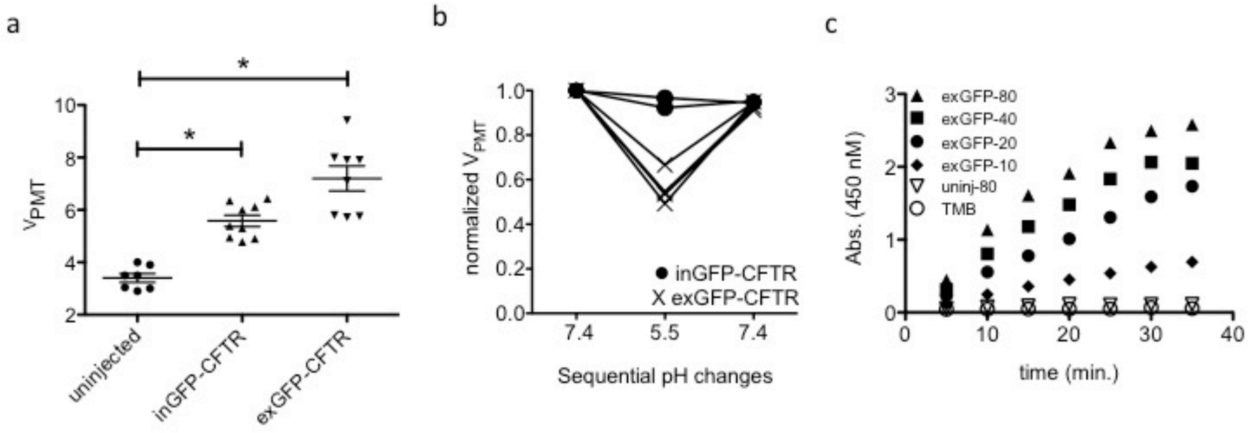

d

		Fluorescence measurement #1		SMase treatment		Fluorescence measurement #2		
pH	7.4	5.5	7.4	7.4	7.4	5.5	7.4	7.4
Treatment	none	none	IBMX	IBMX+SMase	IBMX	none	IBMX+GlyH	none
CFTR activation state	inactive	inactive	Activation	Inhibition occurs	inhibited	inhibited	inhibited	inactive
Duration	~ 5 min	~ 30 s	30-60 min	10 min	5 min	30 s	~ 1 min	~ 5 min

---

Supplementary Figure S2 – Controls for voltage clamp fluorometry: (a) Cells expressing either intracellular or extracellular GFP-tagged CFTR variants showed fluorescence levels significantly above that of uninjected cells. (b) Perfusion of cells expressing the extracellularly-tagged GFP construct with an acidic recording solution led to reversible quenching while the fluorescence of cells expressing the intracellularly-tagged construct was unaffected in this time domain. (c) Panel C shows example data from the cell-ELISA experiment in which we probed for exGFP-CFTR at the cell membrane in unpermeablized cells with an anti-GFP antibody. Accumulation of colorimetric substrate was proportional to the number of exGFP-CFTR expressing cells in the well (in figure,

exGFP-#) demonstrating that the assay absorbance is sensitive to the density of channels in the well and does not arise from nonspecific binding of the anti-GFP primary antibody or the HRP-tagged secondary antibody to the oocyte membrane (see uninjected control). (d) A table showing the VCF experimental design.

Supplementary Fig. S3

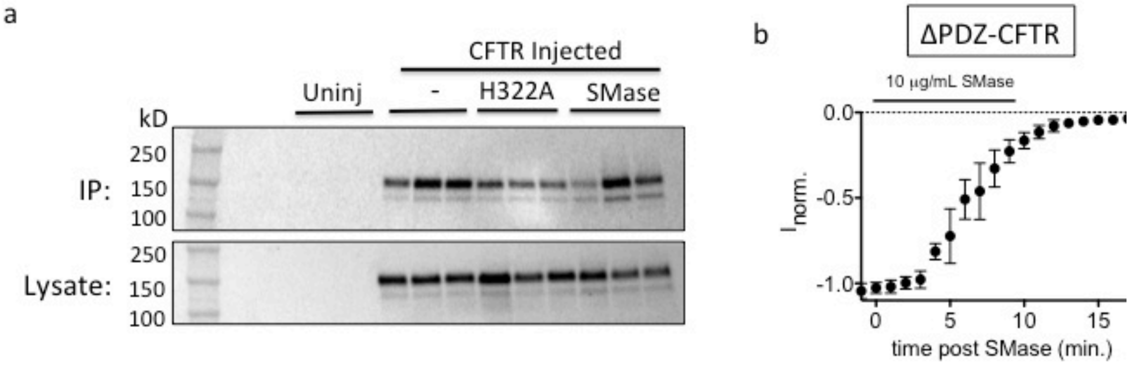

Supplementary Figure S3 – SMase treatment does not affect biotinylation of WT CFTR: (a) Oocytes expressing WT CFTR were either untreated, treated with 10 µg/mL H322A SMase, or treated with 10 µg/mL WT SMase for 10 minutes before exposure to the membrane impermeant biotinylation reagent Sulfo-NHS-SS-biotin. This treatment would be expected to decrease CFTR currents by ≥95%; however, it did not significantly change labeling of CFTR. Each lane represents an experimental replicate and uninjected oocytes (“uninj”) were used as an additional negative control. (b) A summary TEVC trace showing inhibition of CFTR lacking the C-terminal PDZ domain (DTRL, ΔPDZ-CFTR) by 10 µg/mL SMase (n=3).

Supplementary Fig. S4

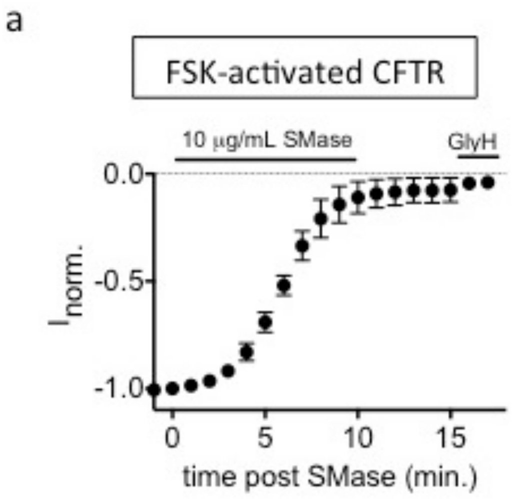

Supplementary Figure S4 – FSK-activated WT-CFTR and split-ΔR-CFTR are sensitive to SMase-mediated inhibition: (a) A summary TEVC trace showing inhibition of WT-CFTR following activation with 10 μM FSK alone, indicating that potential direct binding of IBMX to CFTR does not lead to the appearance of a state sensitive to SMase. Similarly the presence of FSK is not leading to the result reported by Ramu et al. (n=3). (b) A summary TEVC trace shows inhibition of split-ΔR-CFTR by 10 μg/mL SMase (n=3).

Supplementary Fig. S5

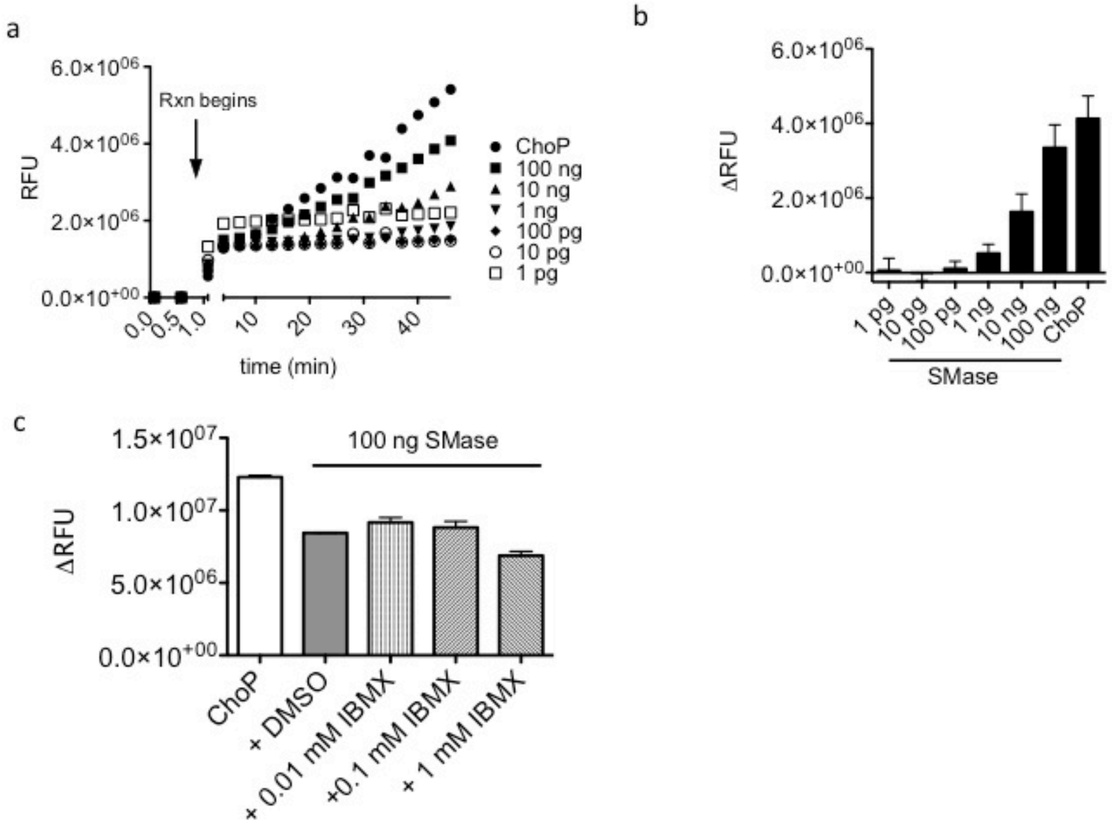

Supplementary Figure S5 – IBMX does not directly inhibit SMase: (a) Representative data showing the time-dependent accumulation of resorufin fluorescence in the presence of increasing masses of purified SMase using the fluorometric SMase assay described in Methods. Phosphocholine (ChoP) is used as a positive control and to define maximal signal. (b) Cumulative data showing that the fluorescence signal is proportional to the mass of SMase added (n=2-4). (c) Addition of IBMX did not significantly affect SMase activity as compared to the DMSO vehicle control (1-way ANOVA with Dunnett's multiple comparison test, n=2-4).

Supplementary Fig. S6

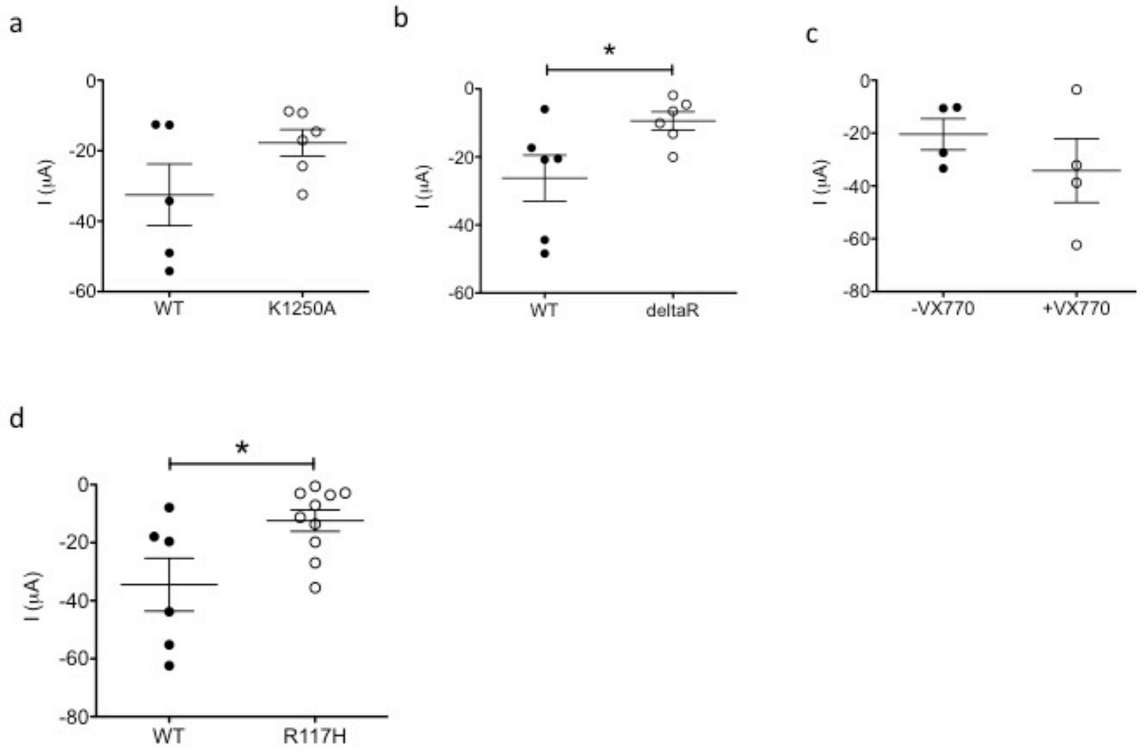

<u>Supplementary Figure S6 – Differential sensitivity of gating mutants does not result from altered expression levels</u>: (a) Total current magnitude of WT and K1250A CFTR expressing cells was not different. (b) Cells expressing WT CFTR showed significantly more current than cells expressing split-ΔR-CFTR, but WT CFTR currents were more sensitive to SMase (Fig. 5b). (c) Cells treated with VX-770 did not have significantly more current than untreated cells. This was achieved by reducing expression level in VX-770 treated cells. (d) Current magnitude of R117H expressing cells was slightly lower than that of WT cells. Given the gating defects associated with the R117H mutation that reduce the activity of this mutant, this likely represents a similar expression level.

Supplementary Fig. S7

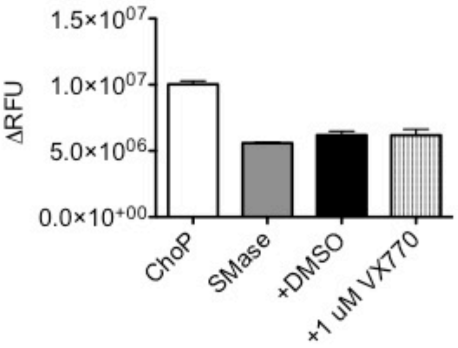

Supplementary Figure S7 – VX-770 does not directly inhibit SMase – SMase was not significantly inhibited by 1 µM VX-770 in the Amplex Red-based fluorometric assay (1-way ANOVA with Dunnett's multiple comparison test, n=4).

Supplementary Fig. S8

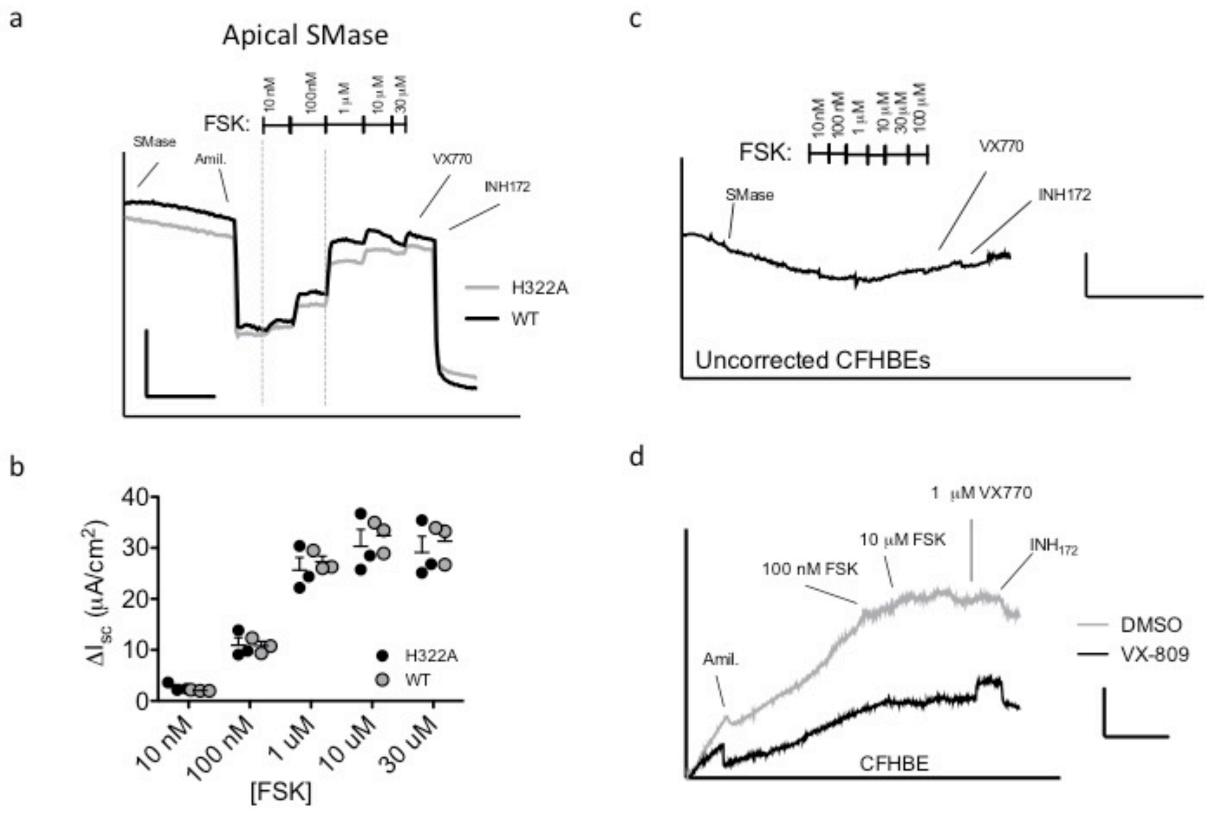

<u>Supplementary Figure S8 – Apical SMase does not affect NHBE currents and CFHBEs do not show VX-770-stimulated currents</u>: (a) Example trace showing similar activation of transepithelial current in NHBEs pretreated with either WT or H322A SMase apically. Scale bars represent 20 μA/cm^2 and 20 mintues. (b) Summary data showing that there was no difference in transepithelial currents following apical SMase treatment. (c) CFHBE cells incubated at 37°C do not show currents in the presence of 100 μM FSK and 1 μM VX-770. Scale bars: 2 μA/cm^2 and 50 minutes. (d) Example trace shows that CFHBEs corrected with 3 μM VX-809 showed a similar phenotype to the temperature corrected CFHBEs. Scale bars: 2 μA/cm^2 and 5 minutes

Supplementary Figure S9

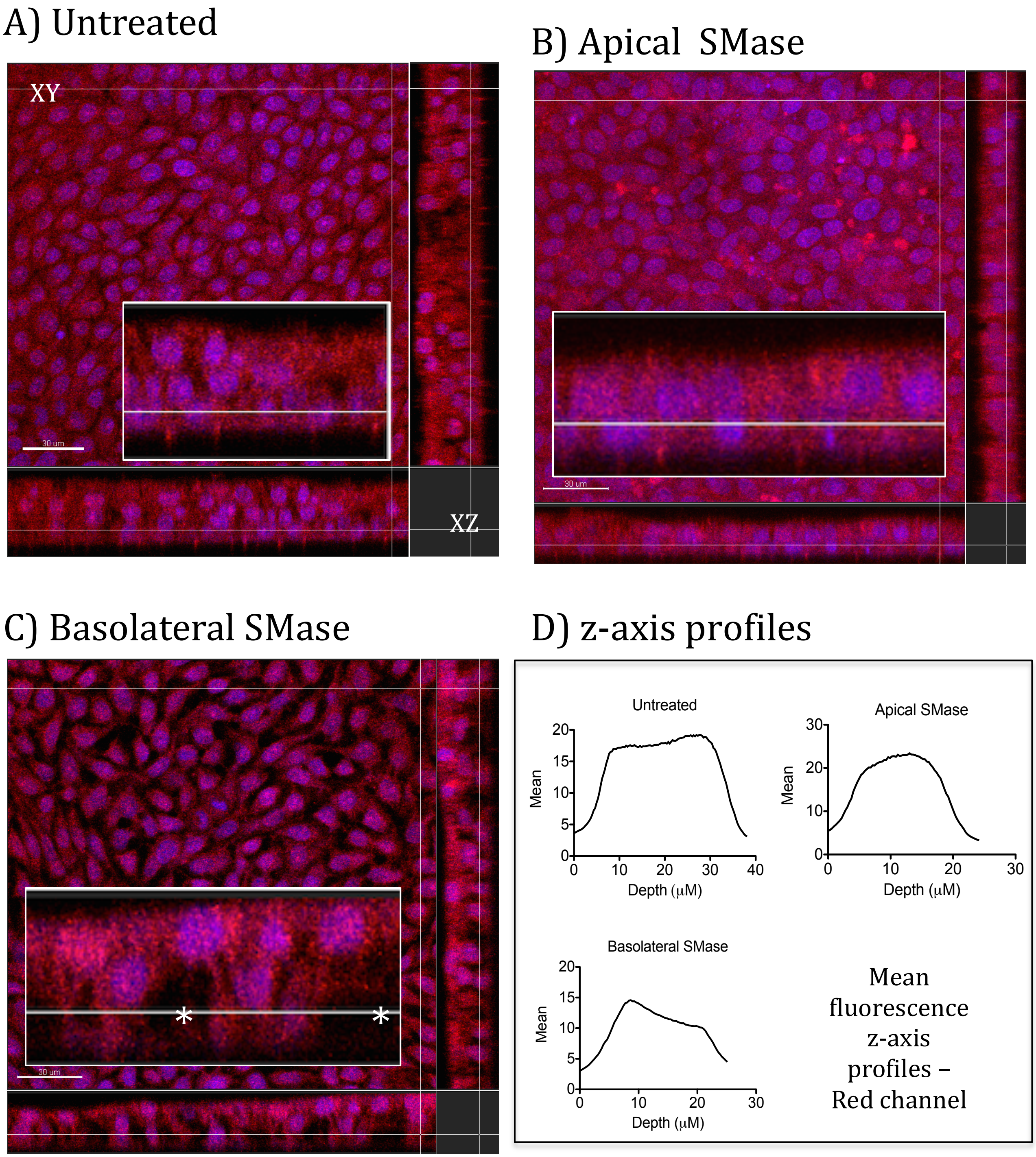

Supplementary Figure S9 - Basolateral but not apical SMase treatment affects sphingomyelin distribution: (a) An example confocal section shows a uniform and continuous distribution of mCherry-NT-lysenin near the basolateral membrane in cells that have not been treated with SMase. White outlined box shows a zoomed xz section. (b) NHBE cells that were treated with apical SMase show a similar pattern of staining suggesting that cellular sphingomyelin is relatively unaffected. White outlined box shows a zoomed xz section. (c) Following basolateral treatment with SMase, there is a clear reduction in basolateral staining of NHBEs by mCherry-NT-lysenin and the appearance of an irregular staining pattern in both the xy and xz planes near the filter (shown). White outlined box shows a zoomed xz section. Two example voids are marked by asterisks. (d) Plot profiles showing the z-axis mean intensity in the red channel over the entire section. “Untreated” plot profile shows clear change in signal corresponding with the apical and basolateral membranes and a similar pattern is seen in the “Apical SMase” treatment group. Conversely, the “Basolateral SMase” group shows a depth dependent reduction in signal apparent as a slow decrease in mean fluorescence intensity in the z-plot between ~10 and 20 μM. This unilateral change in sphingomyelin homeostasis correlates well with the observation reported here and by others that only basolateral SMase treatment affects ion channel function in polarized epithelia. Similar results were seen in 3-4 fields across 2 filters per condition.
